# Hypoxia/Reoxygenation Cardiac Injury and Regeneration in Zebrafish Adult Heart

**DOI:** 10.1371/journal.pone.0053748

**Published:** 2013-01-16

**Authors:** Valeria Parente, Serena Balasso, Giulio Pompilio, Lorena Verduci, Gualtiero I. Colombo, Giuseppina Milano, Uliano Guerrini, Lidia Squadroni, Franco Cotelli, Ombretta Pozzoli, Maurizio C. Capogrossi

**Affiliations:** 1 Laboratorio di Biologia Vascolare e Medicina Rigenerativa, Centro Cardiologico Monzino, Istituto di Ricovero e Cura a Carattere Scientifico, Milan, Italy; 2 Istituto Nazionale Genetica Molecolare, Milan, Italy; 3 Laboratorio di Immunologia e Genomica Funzionale, Centro Cardiologico Monzino, Istituto di Ricovero e Cura a Carattere Scientifico, Milan, Italy; 4 Dipartimento di Scienze Farmacologiche e Biomolecolari, Laboratorio di Farmacologia della Trombosi e dell’Aterosclerosi, Università degli Studi di Milano, Milan, Italy; 5 Divisione di Cardiologia, Ospedale S. Carlo Borromeo, Milan, Italy; 6 Dipartimento di Biologia e Dipartimento di Bioscienze, Laboratorio di Biologia dello Sviluppo, Università degli Studi di Milano, Milan, Italy; 7 Laboratorio di Patologia Vascolare, Istituto Dermopatico dell’Immacolata, Istituto di Ricovero e Cura a Carattere Scientifico, Rome, Italy; Virginia Commonwealth University Medical Center, United States of America

## Abstract

**Aims:**

the adult zebrafish heart regenerates spontaneously after injury and has been used to study the mechanisms of cardiac repair. However, no zebrafish model is available that mimics ischemic injury in mammalian heart. We developed and characterized zebrafish cardiac injury induced by hypoxia/reoxygenation (H/R) and the regeneration that followed it.

**Methods and Results:**

adult zebrafish were kept either in hypoxic (H) or normoxic control (C) water for 15 min; thereafter fishes were returned to C water. Within 2–6 hours (h) after reoxygenation there was evidence of cardiac oxidative stress by dihydroethidium fluorescence and protein nitrosylation, as well as of inflammation. We used Tg(cmlc2:nucDsRed) transgenic zebrafish to identify myocardial cell nuclei. Cardiomyocyte apoptosis and necrosis were evidenced by TUNEL and Acridine Orange (AO) staining, respectively; 18 h after H/R, 9.9±2.6% of myocardial cell nuclei were TUNEL^+^ and 15.0±2.5% were AO^+^. At the 30-day (d) time point myocardial cell death was back to baseline (n = 3 at each time point). We evaluated cardiomyocyte proliferation by Phospho Histone H3 (pHH3) or Proliferating Cell Nuclear Antigen (PCNA) expression. Cardiomyocyte proliferation was apparent 18–24 h after H/R, it achieved its peak 3–7d later, and was back to baseline at 30d. 7d after H/R 17.4±2.3% of all cardiomyocytes were pHH3^+^ and 7.4±0.6% were PCNA^+^ (n = 3 at each time point). Cardiac function was assessed by 2D-echocardiography and Ventricular Diastolic and Systolic Areas were used to compute Fractional Area Change (FAC). FAC decreased from 29.3±2.0% in normoxia to 16.4±1.8% at 18 h after H/R; one month later ventricular function was back to baseline (n = 12 at each time point).

**Conclusions:**

zebrafish exposed to H/R exhibit evidence of cardiac oxidative stress and inflammation, myocardial cell death and proliferation. The initial decrease in ventricular function is followed by full recovery. This model more closely mimics reperfusion injury in mammals than other cardiac injury models.

## Introduction

Recent studies have shown that the adult zebrafish heart, unlike the mammalian heart, exhibits the ability to fully regenerate within weeks after surgical removal of the ventricular apex [1], [2], cryoinjury [3], [4], [5] and genetic cardiomyocyte ablation [6]. Zebrafish has a two-chamber heart constituted by an atrium, a ventricle and a single coronary artery [7]; similar to reptiles’ the zebrafish heart derives its oxygen and nutrients from the blood that bathes the spongy myocardium and partially from coronary artery flow. In the amputation injury model about 20% of the apex is surgically removed; this is followed by the formation of a blood clot and fibrin deposition in the damaged area and, within days, by consistent growth of new cardiomyocytes, with little or no scarring. About three months after amputation, the regeneration process is complete and, at a morphological level, previously damaged hearts are indistinguishable from uninjured hearts [1]. This species peculiarity has made the zebrafish an attractive model to study the mechanisms for heart regeneration following injury. However, mechanical cardiac injury, cryoinjury, and genetic cardiomyocyte ablation lack some of the key elements that cause and are associated with heart damage in mammals following coronary occlusion [3], [4], [5], *e.g.* there is no oxygen deprivation, it is unknown whether free radicals are produced, the cardiac apex is removed and this prevents the development of extensive cell death in the injured area as it occurs in the severely ischemic heart. Therefore, there is a need to develop a zebrafish model of cardiac injury that more closely mimics what occurs in mammals following coronary artery occlusion; such model would be expected to be more suitable to study the mechanisms of damage and regeneration in zebrafish than surgical amputation of the apex, cryoinjury, or genetic cardiomyocyte ablation. In the present work, we developed a hypoxia/reoxygenation (H/R) injury model that affects the zebrafish heart and exhibits characteristics of reperfusion injury in the mammalian heart; acute cardiac damage is followed by spontaneous regeneration and functional recovery.

## Materials and Methods

### Zebrafish Husbandry

The following zebrafish lines were used in the present work: adult zebrafish (*Danio rerio*; AB wild-type strain); the transgenic line Tg(MPO:EGFP)×Tg(LysC:DsRed) (kind gifts of Stephen A Renshaw, University of Sheffield, UK and Chris Hall, University of Auckland, New Zealand, respectively); the transgenic line Tg(cmlc2:nucDsRed) (kind gift of Geoffrey C. Burns, Children’s Hospital, Boston, MA). Yellow-fluorescent (LysC^+^/MPO^+^) and green-fluorescence (predominantly, MPO^+^) cells identify neutrophils and red-fluorescent (LysC^+^) cells identify macrophages; cmlc2^+^ nuclei are red and identify cardiomyocytes. Zebrafish were maintained at 28.5°C in 14∶10 hours light/dark conditions, 50 adults/10 L tank according to standard methods. All procedures adhered to the guidelines from the Italian Ministero della Sanità, Office of the Animals Scientific Procedures, protocol project number N° 09/2009.

### Induction of Hypoxia/Reoxygenation

We used a 5 L glass tank in which water-dissolved O_2_ concentration and pH were monitored by dissolved-oxygen and pH probes respectively (Aquatic Habitat; Apopka, FL). Regular calibration of the probe was performed at room temperature with deionised water, air-saturated at atmospheric pressure. The O_2_ concentration in the tank was lowered by bubbling a mixture of 95% Argon and 5% CO_2_; under these conditions water-dissolved O_2_ gradually decreased from 80% (6.5 mg/L) to 5% (0.4 mg/L). A mixture of Argon and O_2_ was utilized because Argon is heavier than air and, when used to bubble a solution in an open chamber, it forms a gas seal at the surface of the solution, therefore preventing water contamination by O_2_ present in room air [8], [9]. NaHCO_3_ was added to the water in order to keep pH at 7.50 and avoid acidification during hypoxia. Under these experimental conditions hypoxia induces intracellular acidification in spite of a physiologic extracellular pH [10]. This experimental condition more closely mimics what occurs in the initial phases of ischemia, i.e. extracellular pH is still within the physiologic range and lactic acid has not yet been released by the cells. Extracellular and systemic acidification occur in a more advanced phase of ischemia. Adult 4–6 month-old zebrafish were transferred into the tank containing water with 5% O_2_ concentration, following equilibration with 95% Argon and 5% CO_2_, and kept in it for 15 min. Under these conditions mortality was ∼1%; it is noteworthy that in preliminary experiments we found that longer exposure times to hypoxic water were associated with a significant increase in zebrafish mortality (data not shown). After hypoxia zebrafish were immediately transferred to normoxic water, 1 adult in individual 1 L tank. Upon returning to normoxia fishes appeared stunned and it took them approximately 10 minutes to recover a normal behaviour and resume normal swimming. The animals were sacrificed at different time points after H/R and the heart was explanted. Untreated zebrafish kept under normoxic conditions were used as controls. Hearts for gene expression and immunoblot analysis were immediately snap-frozen in liquid nitrogen. For histology and microscopy studies hearts were embedded in OCT or paraffin. Cryosections, 8µm thickness, were cut with a cryostat (Microm HM560; Thermo Scientific Microm, Walldorf, Germany) at −20°C and transferred onto SuperFrost-Plus slides (Thermo Scientific Menzel-Glaser, Braunschweig, Germany). Paraffin sections, 5µm thickness, were cut with a microtome (RM2245; Leica, Wetzlar, Germany), and processed according to standard procedures.

### Measurement of Intracellular Reactive Oxygen Species After H/R

Superoxide anion (O_2_^–^) was detected in zebrafish freshly cut heart sections by DHE (Dihydroethidium) staining. DHE (Invitrogen, Carlsbad, CA), an oxidative fluorescent probe, was used to detect reactive oxygen species (ROS) in treated heart sections 2 h, 6 h and 14 h after H/R. Normoxic fishes were used as controls. In the presence of superoxide anion radical, DHE is converted to the fluorescent molecule ethidium which labels nuclei by intercalating DNA.

In order to determine proteins nitrosylation, immunofluorescence staining was performed with an anti-nitrotyrosine (N-Tyr) polyclonal antibody (Calbiochem, San Diego, CA) on cryosections (see details in Data S1).

### HIF-1α – dependent Genes Expression and Determination of Cardiac Cell Apoptosis

Total RNA was isolated from whole adult heart in order to measure by Real-Time PCR the expression level of HIF-1α-dependent gene. In order to detect cardiac cell apoptosis in the whole heart, three independent methods were used: detection of cytosolic oligonucleosome-bound DNA by ELISA, quantitation of caspase-3 activity by immunoblotting and *in situ* detection of DNA fragmentation by TUNEL assay (CardioTACS, Trevigen). These experiments were performed according to standard protocols, and details can be found in the Methods section in Data S1.

### Determination of Myocardial Cell Death and Proliferation

Myocardial cell apoptosis, necrosis and proliferation were evaluated in heart cryosections (8µm) from Tg(cmlc2:nucDsRed) untreated or H/R zebrafish. Cardiomyocyte apoptosis was evaluated with the *In Situ* Cell Death Detection Kit, Fluorescein (Roche Applied Science), following manufacturer's protocol. Myocardial cell necrosis was evaluated by Acridine Orange (AO) staining, according to the standard protocol [5], [11], [12]. Cardiomyocyte proliferation was assessed by immunofluorescence staining with Phospho Histone H3 (pHH3) and Proliferating Cell Nuclear Antigen (PCNA) markers.

These protocols as well as the procedure we used to quantify cardiomyocyte death and proliferation are described in detail in the Methods in Data S1.

### Cardiac Imaging by Echocardiography

In order to assess cardiac function in normoxic and H/R zebrafish, animals were anesthetized with low-dose tricaine solution (0.04 mg/mL) and placed in a Petri dish filled with a custom-made sponge, with the ventral side upward. The Petri dish was filled with tricaine medium. Two-dimensional (2D) high-resolution real-time *in vivo* images were obtained with the Vevo770 Imaging System (VisualSonics, Toronto, Canada), through a 50–70 MHz scanhead. The ventricle was visualized in B-mode modality in a longitudinal plane. The epicardial border was traced in long-axis views from the atrio-ventricular valve annulus to the apex, then back to the annulus, at end-diastole (ED) and end-systole (ES). Diastolic area (DA), systolic area (SA) and fractional area change (FAC) were measured; FAC was calculated as follows, (DA-SA)/DA*100. Echocardiograms were evaluated by two independent examiners blind to the treatment protocol.

### Statistical Analysis

All results are expressed as mean ± SEM. Differences between control and treatment groups were assessed by One-way analysis of variance (ANOVA) followed by Dunnett’s test for multiple comparisons, or by Kruskal-Wallis test followed by Dunn's multiple comparison test, when appropriate. Differences in echocardiographic parameters were evaluated by Repeated measures ANOVA followed by Dunnett’s test. A *p*-value <0.05 was considered statistically significant.

## Results

### Hypoxia/reoxygenation Induces Oxidative Stress and Inflammation in Zebrafish Heart

We first examined whether H/R induces oxidative stress and an inflammatory response in zebrafish adult heart as it is known to do in mammalian heart.

**Figure 1:**
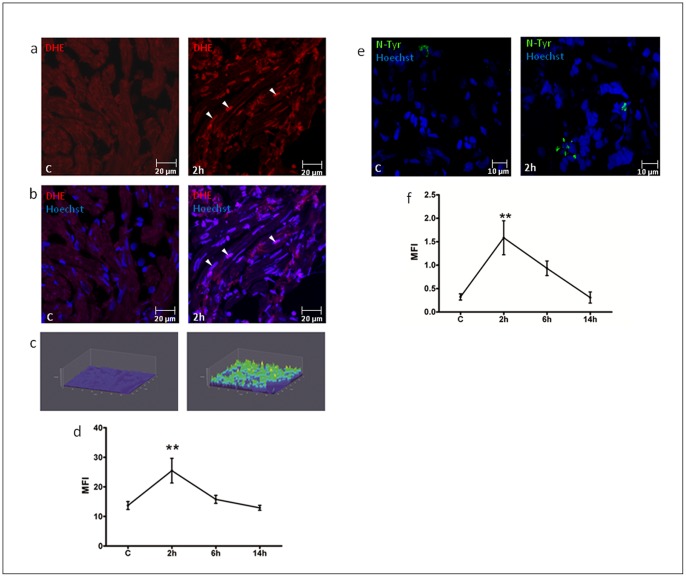
Oxidative stress detection after H/R *in vivo.* DHE staining (a-d) and N-Tyr immunofluorescence (e-f) of hearts under control conditions (C) and exposed to H/R. (a) Representative confocal microscopy images of DHE staining in C and 2 h after H/R. (b) Merge of DHE and Hoechst nuclear staining. Calibration bar = 20 µm. White arrow-heads indicate DHE^+^ nuclei. (c) 3D representation of DHE fluorescence intensity distribution in the analyzed area: the *z-axis* shows the fluorescence intensity in cardiac nuclei, the *y-axis* and *x-axis* show the spatial distribution of nuclei on a plane. (d) Graph shows Mean Fluorescence Intensity (MFI) in C and 2 h to 14 h after H/R (n = 4 at each time point; ** *p*<0.01 *vs.* C). Time course analysis revealed a peak of oxidative stress at 2 h in zebrafish adult heart sections, detected by DHE staining. (e) Representative confocal microscopy images of N-Tyr immunofluorescence, where green fluorescence indicates anti-N-Tyr and Hoechst nuclei staining: control (C, left panel) and 2 h after H/R (right panel). Calibration bar = 10µm. (f) Graph shows Mean Fluorescence Intensity (MFI) in C and 2 h to 14 h after H/R (n = 4 at each time point; ** *p*<0.01 *vs.* C). H/R induced protein nitrosylation with a peak effect at the 2 h time point.

Zebrafish were sacrificed at different times after H/R and the hearts were stained with DHE; upon reacting with superoxide anion DHE is oxidized to ethidium and releases fluorescent red light (see Methods and methods in Data S1). A time course analysis revealed that peak oxidative stress occurred 2 h after H/R and was back to baseline at later time points, *i.e.* at 6 h and 14 h (Fig. 1a-d; S1a-c). In order to confirm H/R-induced ROS production, it was evaluated N-Tyr synthesis in heart sections from the same hearts used for DHE staining. Significant N-Tyr accumulation was detected 2 h after treatment, progressively decreased thereafter and was back to baseline at the 14 h time point (Fig. 1e, f). Therefore, by two different techniques it was found that H/R induced oxidative stress with a peak effect at the 2 h time point.

**Figure 2:**
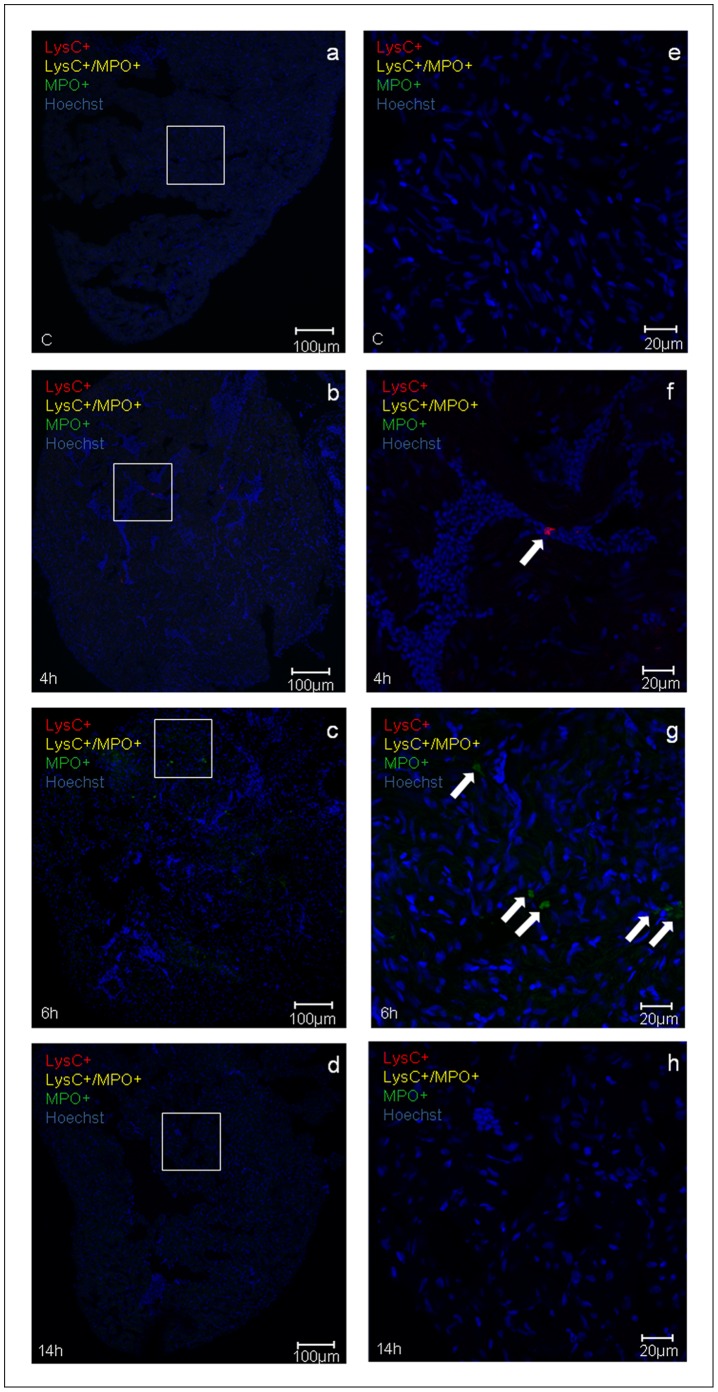
Inflammatory response induced by H/R *in vivo.* Representative confocal microscopy images (a-h) showing neutrophils (yellow fluorescence or green fluorescence) and macrophages (red fluorescence) infiltration in double transgenic line Tg(MPO:EGFP)×Tg(LysC:DsRed) in control (C) and at different time points (4 h, 6 h, and 14 h) after H/R. Neutrophils are either yellow (LysC^+^/MPO^+^) or green (predominantly, MPO^+^) cells (arrows in the 6 h image); red macrophages are LysC^+^ cells (arrow in the 4 h image). Hoechst stains cell nuclei; (a-d) calibration bar = 100 µm, (e-h) calibration bar = 20 µm.The peak inflammatory response occurred at the 6 h time point after H/R This experiment was performed three times with similar results.

In other experiments it was examined whether H/R induced an inflammatory response characterized by neutrophils and macrophages infiltration of the heart. These studies were carried out in the transgenic line Tg(MPO:EGFP)×Tg(LysC:DsRed); in this cross between the two transgenic lines, yellow-fluorescent (LysC^+^/MPO^+^) and green-fluorescence (predominantly, MPO^+^) cells identify neutrophils and red-fluorescent (LysC^+^) cells identify macrophages. Sparse inflammatory cells were observed 4 h after H/R and the peak inflammatory infiltration was evident at the 6 h time point. Infiltration of neutrophils and macrophages rapidly decreased thereafter and was back to baseline 24 h after H/R (Fig. 2). At these time-points, no inflammatory infiltration was detected in the brain of the same zebrafish adults (Fig. S2), whereas neutrophils and macrophages clusters were found in the liver starting from 4 h until 14 h after H/R (Fig. S3).

### Hypoxia/reoxygenation Modulates HIF-1α–dependent Genes in Zebrafish Heart

**Figure 3:**
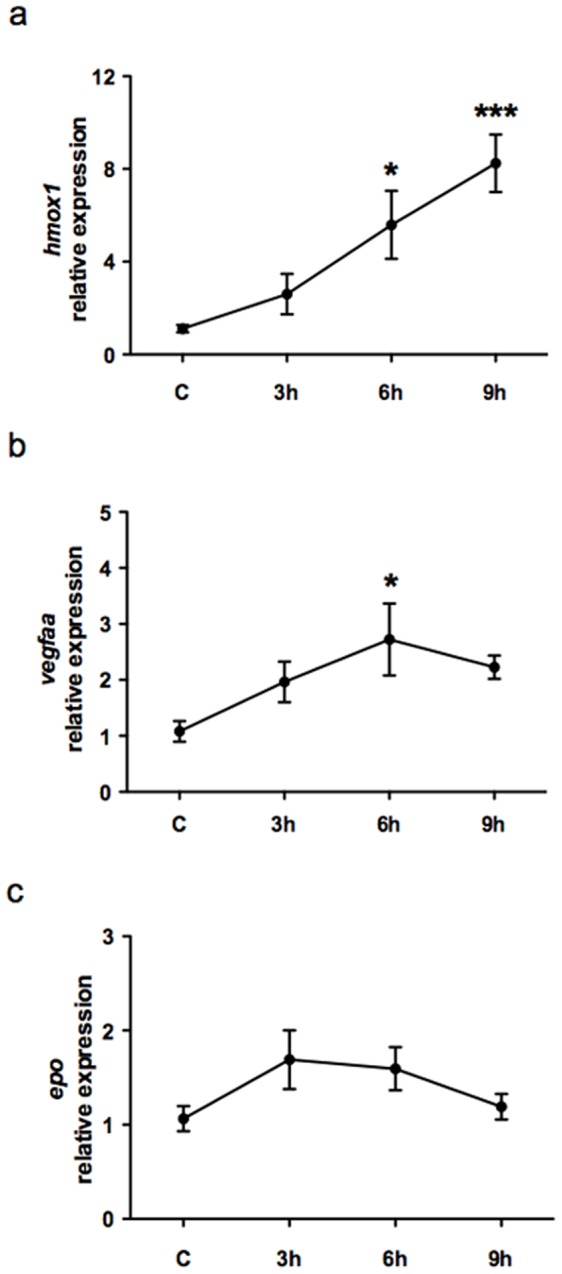
Detection of HIF-1α-dependent genes expression in whole hearts after H/R *in vivo.* Graphs show selected HIF-1α-dependent genes expression in whole hearts in control (C) and at different time points (3 h, 6 h, and 9 h) after H/R. (a) *Hmox1* mRNA expression exhibited a progressive increase and, at the 9 h time point, *hmox1* was ∼8-fold higher than in C. (b) *Vegfaa* mRNA increased and achieved its peak 6 h after H/R. (c) *Epo* mRNA exhibited a peak increase at 3 h which was ∼1.7-fold higher than in C but failed to achieve statistical significance. (n = 6; * *p*<0.05 and *** *p*<0.001 *vs.* C).

To assess whether H/R enhanced hypoxia inducible factor 1α (HIF-1α)–dependent genes, we determined the expression level of *hmox1*, *vegfaa*, and *epo* in the heart at 3 h, 6 h, and 9 h after H/R [13], [14], [15]. *Hmox1* (heme-oxygenase (decycling) 1) mRNA expression progressively increased and, at the 9 h time point, was ∼8-fold higher than control (Fig. 3a). *Vegfaa* (vascular endothelial growth factor Aa) mRNA raised and achieved a plateau 6 h after H/R: the peak increase was ∼2.5-fold control (Fig. 3b). *Epo* (erythropoietin) mRNA showed a peak increase at 3 h, which was ∼1.7-fold control but was not statistically different from baseline (Fig. 3c).

### Hypoxia/reoxygenation Induces Cardiac Cell Apoptosis in Zebrafish Heart

In these experiments different and complementary approaches were taken to establish whether H/R induced cardiac cell apoptosis. Histone-associated DNA fragments detection in the cytoplasm is linked to internucleosomal degradation of genomic DNA occurring during apoptosis. Determination of mono- and oligo-nucleosomes in the cytoplasmic fraction of cardiac tissue lysates was performed; histone-associated DNA fragments were ∼6–7-fold higher than in normoxic control 14 h to 18 h after H/R (Fig. S4a and Table S1a), and their value was back to baseline at the 24 h time point. Since activation of caspases plays a fundamental role in the execution-phase of cell apoptosis [16], [17], we examined the effect of H/R on caspase-3; this is an extensively studied apoptotic protein and its activation by proteolytic cleavage is considered an indicator of cell apoptosis [18]. Caspase-3 activation was evaluated by immunoblot analysis of whole heart lysates and it was found that its peak increase occurred at the 14 h time point, when it was ∼2.8-fold higher than control; thereafter caspase-3 activation exhibited a progressive decrease toward baseline (Fig. S4b and Table S1b). Further, DNA fragmentation was evaluated by TUNEL staining. Apoptotic, i.e. TUNEL^+^, nuclei were quantified at different times after H/R; the peak increase in apoptotic cells was ∼5-fold control and occurred at the 14 h time point; thereafter there was a progressive decrease in TUNEL^+^ nuclei which were back to baseline at the 24 h time point (Fig. S4c and Table S1c). Taken together these experiments show that, under our experimental conditions, H/R induces cardiac cell apoptosis in adult zebrafish heart and that the peak effect on cell death is apparent 14–18 h after reoxygenation.

### Hypoxia/reoxygenation Induces Ventricular Dysfunction in Zebrafish Heart

**Figure 4:**
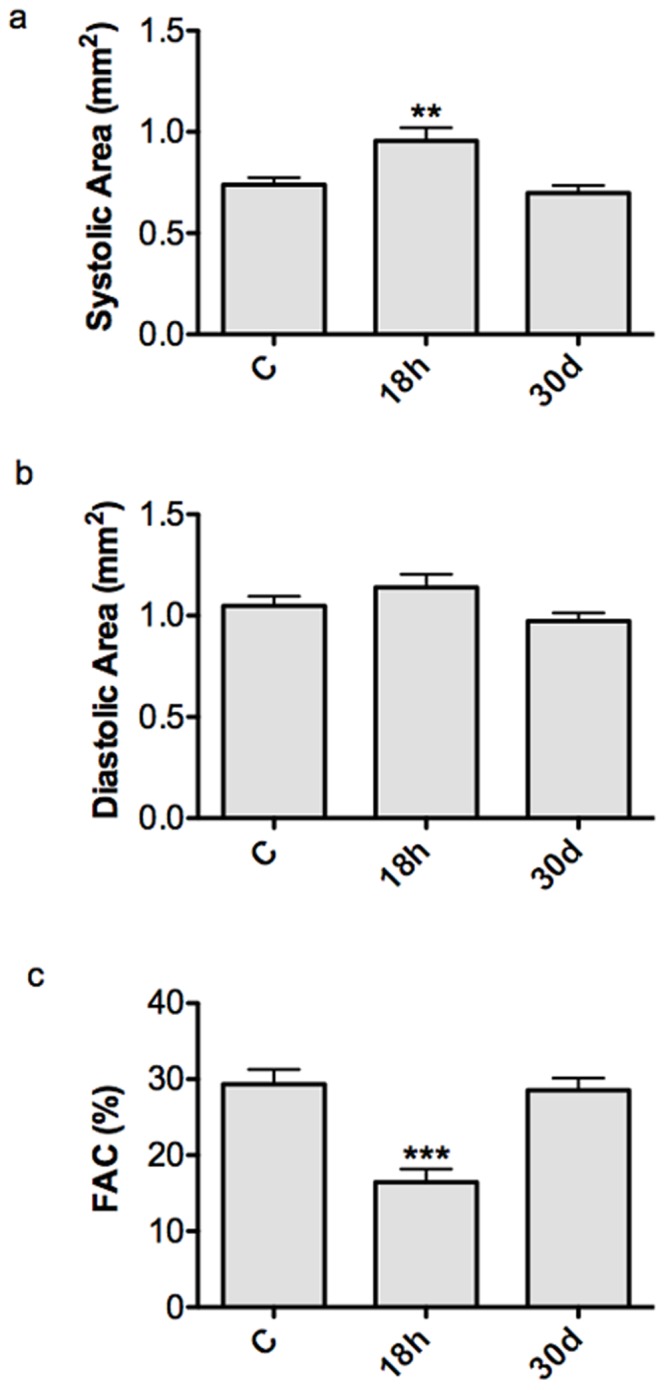
Cardiac function by 2D-echocardiography. Graphs show systolic ventricular area (a), diastolic area (b) and FAC (c) under control conditions (C) and at different time points (18 h and 30d) after H/R. At 18 h after H/R there was a significant increase in SA and decrease in FAC compared to C, which exhibited a full recovery at the 30d time point (** *p*<0.01 and ***p<0.001 *vs.* C). The same animals (n = 12) were used at the different time points.

Cardiac function was assessed by 2D-echocardiography and each animal was analysed before, 18 h and 30d after H/R. It was found that 18 h after H/R there was an increase in SA, from 0.74±0.04 to 0.96±0.07 mm^2^ (*p*<0.01) (Fig. 4a; Table S2) and DA from 1.05±0.05 to 1.14±0.07 mm^2^ (Fig. 4b; Table S2); FAC decreased from 29.3±2.0% in normoxia to 16.4±1.8% (*p*<0.001) (Fig. 4c; Table S2). SA and FAC fully recovered to control value 30d after H/R and they were 0.70±0.04 mm^2^ and 28.6±1.6%, respectively. Movies of representative echocardiograms of control hearts and of hearts exposed to H/R, 18 h and 30d after treatment, are available online in the (movie S1, control; movie S2, 18 h after H/R; movie S3, 30d after H/R).

In additional experiments it was examined whether the functional changes described above were associated with cardiomyocytes death and proliferation.

### Hypoxia/reoxygenation Induces Myocardial Cell Death in Zebrafish Heart

**Figure 5:**
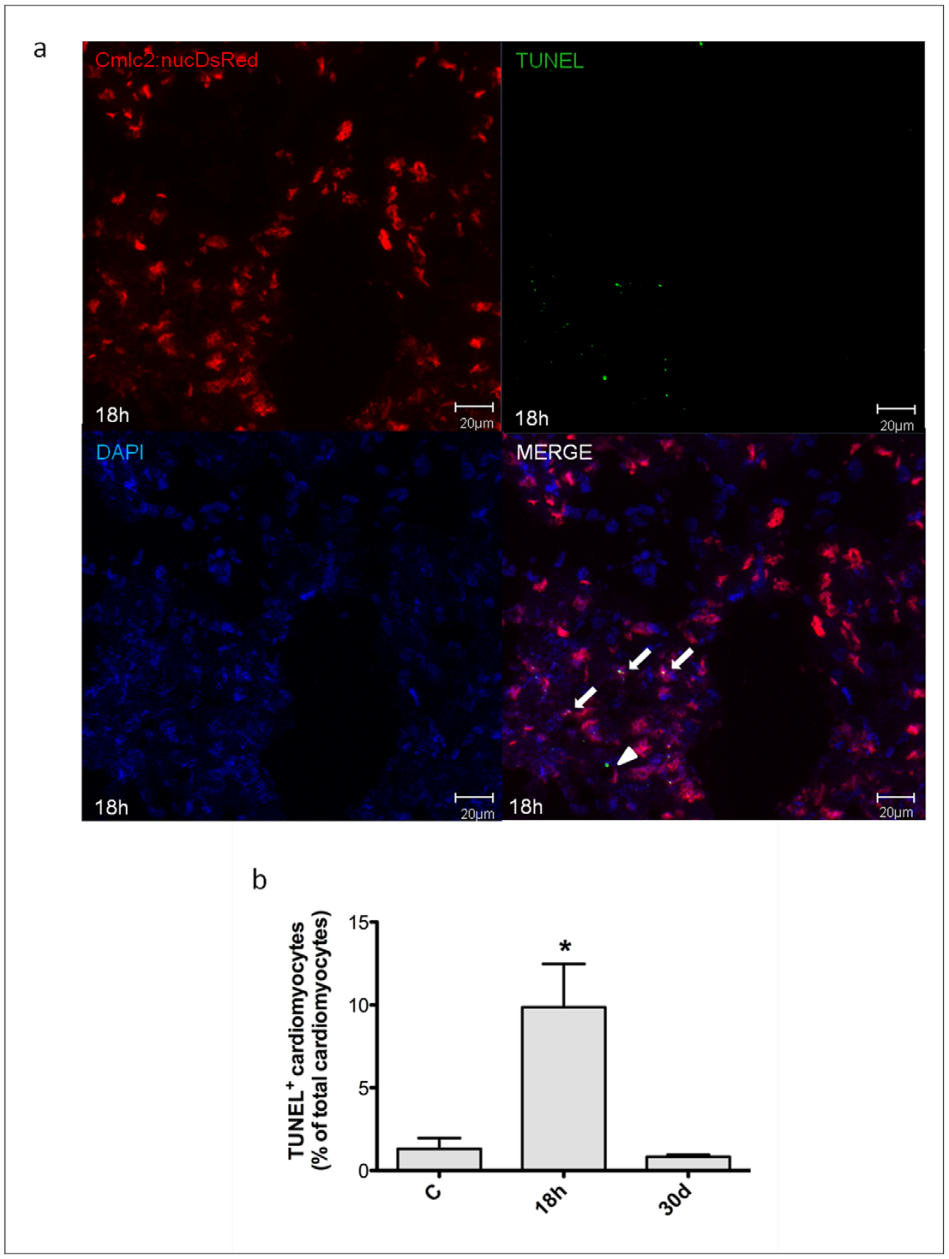
Apoptotic myocyte cell death induced by H/R *in vivo.* Apoptotic myocyte cell death was assessed under baseline conditions, and 18 h and 30d after H/R in the Tg(cmlc2:nucDsRed) zebrafish line. At 18 h after H/R it was found a marked increase in apoptotic myocardial cell number, which was back to control value at the 30d time point. (a) Representative image of a zebrafish heart ventricular section 18 h after H/R, showing colocalization of DAPI, DsRED and TUNEL stainings. Arrows indicate cardiomyocyte TUNEL^+^ nuclei, whereas arrow-head indicates non-cardiomyocyte TUNEL^+^ nuclei. (b) TUNEL^+^ cardiomyocytes nuclei in control (C) animals, and 18 h and 30d after H/R (n = 3 at each time point; * *p*<0.05 *vs.* C).

**Figure 6:**
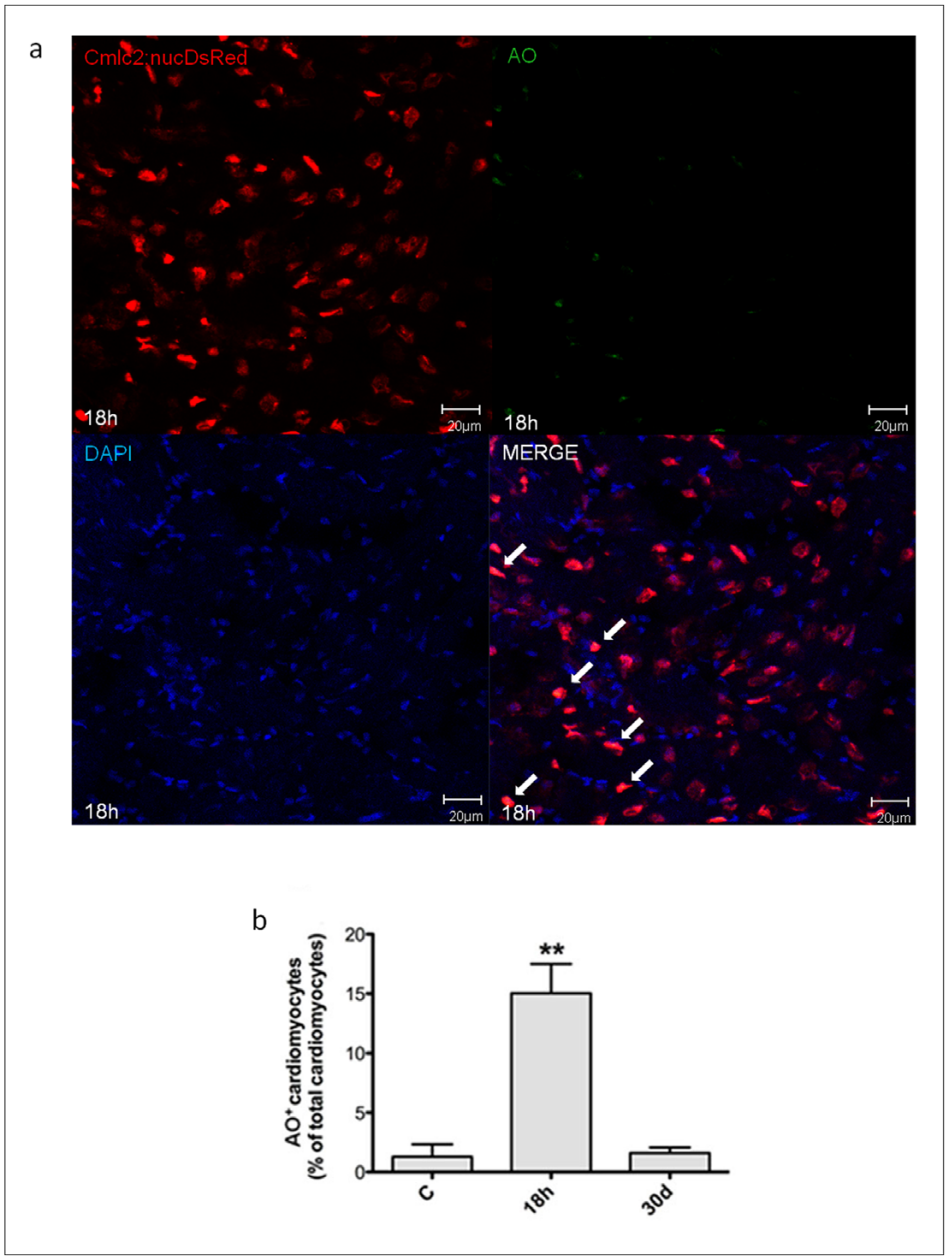
Necrotic myocyte cell death induced by H/R *in vivo.* Necrotic myocyte cell death was assessed under baseline conditions, and 18 h and 30d after H/R in the Tg(cmlc2:nucDsRed) zebrafish line. At 18 h after H/R it was found a marked increase in necrotic myocardial cell number, which was back to control value at the 30d time point. (a) Representative image of a zebrafish heart ventricular section 18 h after H/R showing colocalization of DAPI, DsRED and AO stainings. Arrows indicate cardiomyocyte AO^+^ nuclei. (b) AO^+^ cardiomyocytes nuclei in control (C) animals, and 18 h and 30d after H/R (n = 3 at each time point; ** *p*<0.01 *vs.* C).

In these experiments it was used the transgenic line Tg(cmlc2:nucDsRed) and cardiomyocyte nuclei were identified by the expression of DsRed. TUNEL assay was performed on 8µm cryosections from untreated zebrafish and animals exposed to H/R. We found that 18 h after H/R 9.9±2.6% of myocardial cell nuclei were TUNEL^+^ and the apoptotic index was ∼8-fold higher than control; however, 30d later the apoptotic index was comparable to that found in control conditions (Fig. 5). Necrotic myocardial cells were identified by AO staining; 18 h after H/R 15.0±2.5% cardiomyocytes exhibited evidence of necrosis, a ∼12-fold increase *vs* control; this value was back to baseline by the 30d time point (Fig. 6). In summary, 18 h after H/R it was found a marked increase both in apoptotic and necrotic myocardial cell number which was back to control at the 30d time point; these data are in agreement with the echocardiographic results which showed an initial decrease followed by full recovery of ventricular function.

### Hypoxia/reoxygenation Induces Myocardial Cell Proliferation in Zebrafish Heart

**Figure 7:**
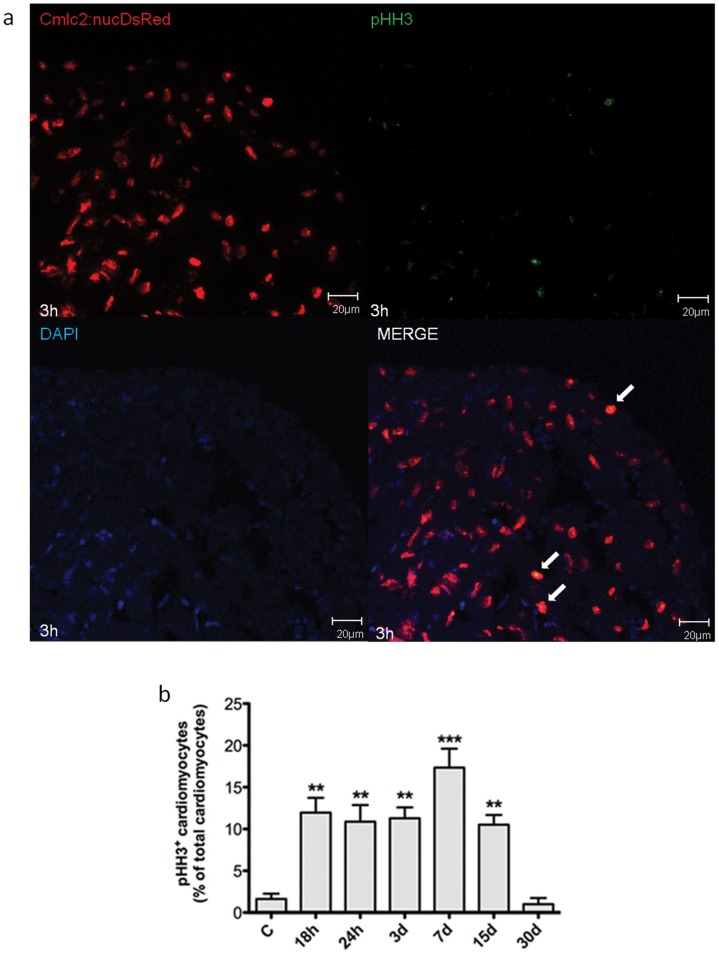
Myocardial cells positive for pHH3 induced by H/R *in vivo.* Cardiomyocytes proliferation was assessed under baseline conditions and 18 h to 30d after H/R in Tg(cmlc2:nucDsRed) zebrafish line. (a) Representative image of a zebrafish heart ventricular section 3d after H/R showing colocalization of DAPI, DsRED and pHH3 stainings. Arrows indicate cardiomyocyte pHH3^+^ nuclei. (b) The increase in pHH3^+^ cardiomyocytes was apparent 18 h after H/R, achieved its peak at the 7d time point and was back to baseline at the 30d time point (n = 3 at each time point; ** *p*<0.01 and *** *p*<0.001 *vs.* C).

**Figure 8:**
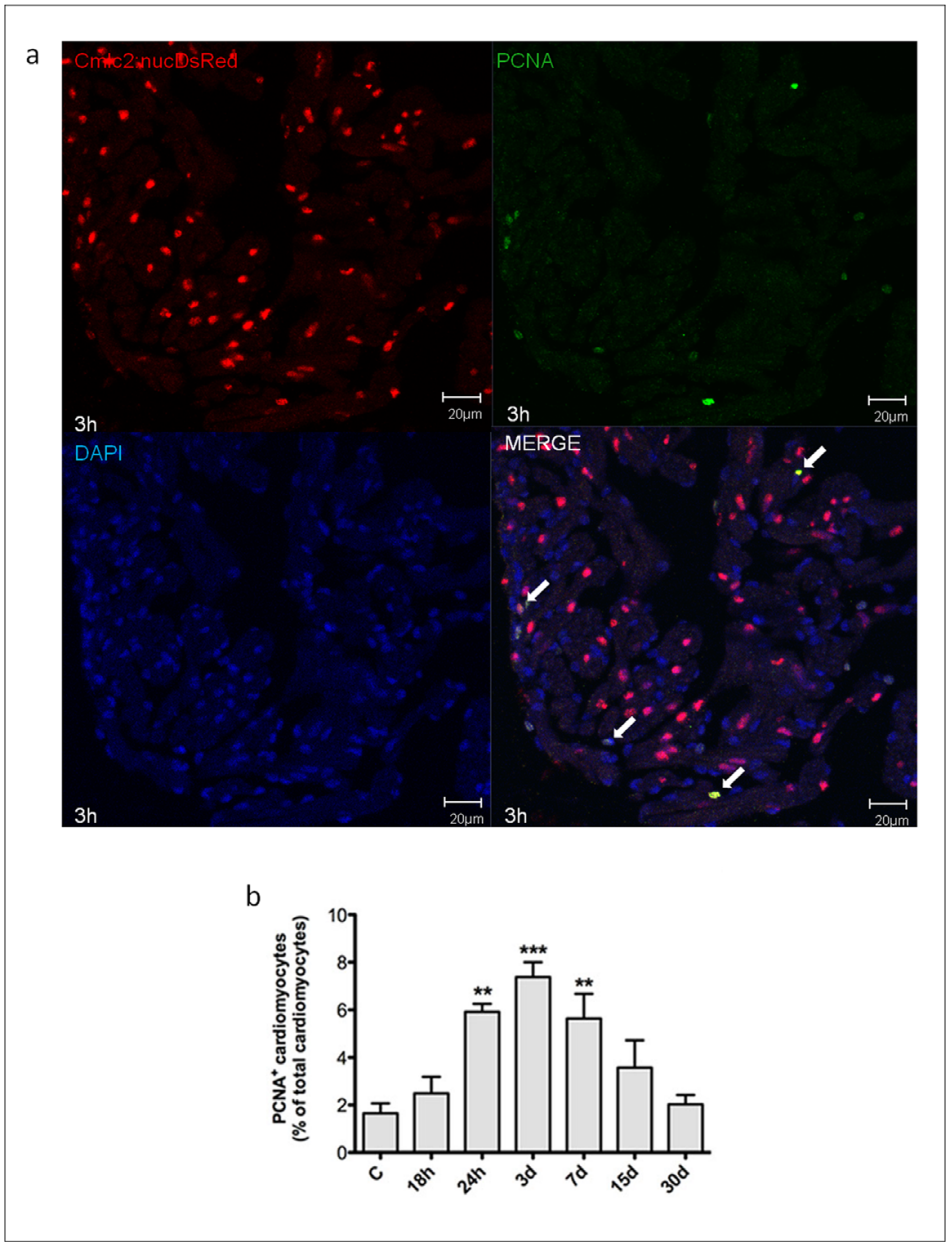
Myocardial cells positive for PCNA induced by H/R *in vivo.* Cardiomyocytes proliferation was assessed under baseline conditions and 18 h to 30d after H/R in Tg(cmlc2:nucDsRed) zebrafish line. (a) Representative image of a zebrafish heart ventricular section 18 h after H/R showing colocalization of DAPI, DsRed and PCNA stainings. Arrows indicate cardiomyocyte PCNA^+^ nuclei. (b) Following H/R, there was a progressive increase in PCNA^+^ cardiomyocytes nuclei; the peak increase was achieved at the 3d time point, and at 30d the number of PCNA^+^ myocardial cells was back to control value (n = 3 at each time point; ** *p*<0.01 and *** *p*<0.001 *vs.* C).

In the following experiments it was examined H/R effect on cardiomyocyte proliferation in the transgenic line Tg(cmlc2:nucDsRed) by using two different markers, the mitotic marker pHH3 (Fig. 7) and PCNA (Fig. 8) which, similarly to BrdU, labels DNA synthesis during mitosis. By pHH3 staining we found evidence of myocardial cell proliferation at the 18 h time point and the peak in pHH3^+^ cardiomyocytes occurred 7d after H/R (17.4±2.3% of all cardiomyocytes; ∼11-fold increase over normoxic control). Similarly, by PCNA staining there was evidence of myocardial cell proliferation 24 h after H/R and peak expression was achieved at the 3d time point (7.4±0.6% of all cardiomyocytes; ∼4-fold increase over normoxic control). Thereafter, both pHH3 and PCNA staining showed a progressive decrease in proliferating myocytes and a return to baseline by the 30d time point. It is noteworthy that both proliferating and dying cardiomyocytes were detected throughout the ventricle.

Finally, we examined whether H/R induced scar formation. By Masson trichrome staining we found no evidence of collagen deposition, neither in control hearts nor 3d and 30d after H/R (n = 3 at each time point; Fig. S5).

## Discussion

The present work describes a new model of cardiac damage, induced by acute hypoxia/reoxygenation, in adult zebrafish; this model reproduces some of the key features of reperfusion injury in mammals. Zebrafish hearts exposed to H/R exhibit evidence of enhanced oxidative stress, inflammatory response, activation of HIF-1α-dependent genes, myocardial cell apoptosis and necrosis, and depressed ventricular function. Further, H/R also activates a myocardial cell proliferative response associated with full recovery of ventricular function.

It is noteworthy that, over the past 35 years, reperfusion injury has become a major focus of interest in cardiovascular research since spontaneous reperfusion occurs frequently in patients with acute coronary syndromes, and therapeutic revascularization with coronary angioplasty, within hours after coronary occlusion represents the standard of care in the management of patients with acute ischemia. The mechanisms and functional *sequelae* of reperfusion/reoxygenation injury have been extensively characterized in isolated cardiac cells [8], isolated hearts [19], live animals and in patients [20], [21].

Since Poss et al. initial description that the zebrafish adult heart can spontaneously regenerate following surgical removal of ∼20% of the ventricle [1], that model of mechanical damage has been widely used to study the mechanisms that lead to cardiac repair in zebrafish. It has been examined whether the key role in regeneration is played by epicardial progenitor cells [2] or by adult cardiomyocyte dedifferentiation and proliferation [22]. Further, fibroblast growth factor and Platelet-derived growth factor [23], [24], notch signaling [25], transcription factors of the Msx family and *GATA4* expression by proliferating cardiomyocytes [26] have all been shown to be important in heart regeneration.

Recently, independent research groups have described cryoinjury [3], [4], [5] and genetic cardiomyocyte ablation [6] as novel methods to induce cardiac damage in adult zebrafish. These studies have confirmed that zebrafish heart can regenerate after injury, however similarly to cardiac apex amputation, these models do not mimic reperfusion injury. In mammals, reperfusion injury is linked to a burst in ROS production which occurs at the time the ischemic or hypoxic heart is reperfused or exposed to oxygen [27]. Therefore, we first examined whether H/R induced oxidative stress in zebrafish heart. Evidence of ROS production was found both by DHE fluorescence and by N-Tyr nitrosylation; the time course of these oxidative stress-induced changes was similar, with a peak effect at the first time point examined, i.e. 2 h, followed by a progressive decrease. Moreover, during reperfusion white blood cells adhere to the activated endothelium, contribute to ROS production and, via this mechanism, further enhance cell adhesion [28]. In the present work we show that, similarly to what occurs in mammals, neutrophils and macrophages infiltrated the zebrafish heart and the peak inflammatory response occurred at the 6 h time point, i.e. after the peak in ROS production.

Another key event in reperfusion injury is the occurrence of cell death. Under our experimental conditions H/R induced cardiac cell apoptosis as evidenced by quantitation of cytoplasmic oligonucleosome-bound DNA, caspase-3 activation and TUNEL staining; the peak effect was observed 14–18 h after reoxygenation. Further, in Tg(cmlc2:nucDsRed) zebrafish we found evidence of myocardial cell apoptosis and necrosis; at the 18 h time point, ∼10% of cardiomyocytes were TUNEL^+^ and ∼15% of cardiomyocytes were necrotic as evidenced by AO staining. Both the time course and the magnitude of cell death observed in zebrafish are comparable to what has been shown to occur in the mammalian heart [29].

We also examined the effect of H/R on the expression of selected HIF-1α-dependent genes [30] known to exert a protective action in the ischemic heart, *hmox1* [31], *vegfaa* [32] and *epo* [33], [34]. *Hmox1* exhibited the most pronounced increase and 9 h after H/R it was ∼8-fold control and still rising. *Vegfaa* increased ∼2.5-fold and achieved its peak at the 6 h time point; interestingly a similar time course has been shown in rat hearts following ischemic preconditioning [35]. Erythropoietin is highly expressed in the kidney whereas in the heart it is present at very low level; in the present work H/R induced a trend increase in *epo* which was not statistically significant. Taken together these results indicate that H/R in zebrafish heart activates a cardiac protection gene program which may contribute to limiting the extent of heart damage.

It is noteworthy that H/R caused ventricular dilation and a significant decrease in FAC which, at the 18 h time point, was reduced from ∼ 30% to ∼ 15%; interestingly, one month later ventricular size and function had fully recovered. In the present zebrafish H/R model, we observed a robust induction of cardiomyocyte proliferation that peaked 3–7d after acute injury, as detected by pHH3 and PCNA staining, and was back to baseline at the 30d time point, i.e. at the same time ventricular function was back to normal, suggesting that myocardial cell proliferation may have played a role in ventricular function improvement.

Our work presents some limitations imposed by the model system. The zebrafish heart has only one atrium and one ventricle, oxygen diffusion occurs largely through the spongy endocardium and to a smaller extent via a single coronary artery. Further, the zebrafish heart works under low pressure conditions which are expected to reduce oxygen requirement in comparison to what occurs in mammals [7]. Finally, in order to induce cardiac H/R injury, the whole animal was exposed to low oxygen and then reoxygenated. It is likely that this perturbation may have induced ROS production and the “oxygen paradox” also in tissues other than the heart. Indeed, H/R treatment did not affect the brain whereas an inflammatory response was observed in the liver. The second aspect is that reperfusion after ischemia induces both cardiac cell death and stunning, i.e. a transient and fully reversible decrease in contractile function due to sub-lethal cell damage. In mammals, where extensive cardiac regeneration does not occur, the effects of stunning and infarction can be differentiated because infarction is associated with a persistent loss of function whereas the effect of stunning resolves with time if the underlying cause, e.g. a marked decrease in blood flow, is removed. In zebrafish myocardial cell proliferation and complete regeneration [1], [2] occur after injury, therefore our results do not distinguish between stunning and regeneration following infarction. Nevertheless, although we cannot exclude that myocardial cell stunning may have occurred, we found clear evidence of diffuse cardiomyocyte death and regeneration and this is a plausible mechanism for the initial decrease and subsequent recovery in ventricular function.

In summary, we have developed and characterized a zebrafish model of cardiac damage that presents the key features of reperfusion injury in the mammalian heart and that will prove useful in studies of heart regeneration in *Danio rerio*.

## Supporting Information

Figure S1 **Oxidative stress detection by DHE fluorescence after H/R** ***in vivo.*** (a) Representative confocal microscopy images of DHE staining at 6 and 14 h after H/R. (b) Merge of DHE and Hoechst nuclear staining. Calibration bar = 20 µm. White arrow-heads indicate DHE^+^ nuclei. (c) 3D representation of DHE fluorescence intensity distribution in the analyzed area: the *z-axis* shows the fluorescence intensity in cardiac nuclei, the *y-axis* and *x-axis* show the spatial distribution of nuclei on a plane.
(TIF)

Figure S2 **Brain inflammatory response induced by H/R** ***in vivo.*** (a–h) Representative confocal microscopy images showing neutrophils (yellow or green fluorescence) and macrophages (red fluorescence) infiltration in double transgenic line Tg(MPO:EGFP)×Tg(LysC:DsRed) in control (C) and at different time points (4 h, 6 h, and 14 h) after H/R. Hoechst stains cell nuclei. (n = 3). (a–d) calibration bar = 100 µm, (e–h) calibration bar = 10 µm.
(TIF)

Figure S3 **Liver inflammatory response induced by H/R** ***in vivo.*** (a–h) Representative confocal microscopy images showing neutrophils (yellow or green fluorescence) and macrophages (red fluorescence) infiltration in double transgenic line Tg(MPO:EGFP)×Tg(LysC:DsRed) in control (C) and at different time points (4 h, 6 h, and 14 h) after H/R. Hoechst stains cell nuclei. (n = 3). (a–d) calibration bar = 100 µm, (e–h) calibration bar = 10 µm.
(TIF)

Figure S4 **Cardiac cell apoptosis induced by H/R** ***in vivo.*** (a) ELISA-based quantitation of cytoplasmic oligonucleosome-bound DNA in zebrafish whole heart lysates. The graph shows apoptosis in control (C) and at different time points (14 h, 18 h, and 24 h) after H/R (values are normalized to C; n = 4 at each time point; * *p*<0.05 *vs.* C). Enrichment factor is measured as absorbance of treated heart *vs.* absorbance of control heart. (b) Western blot analysis of caspase-3 activation in whole single zebrafish heart lysates in C and 6 h to 24 h after H/R (values are normalized to C; n = 3 at each time point; * *p*<0.05 *vs.* C). Relative expression is referred to densitometric analysis data. (c) DNA fragmentation by TUNEL staining of paraffin embedded heart sections. The graph shows apoptosis in C and 14 h to 24 h after H/R. Data are expressed as percentage of TUNEL^+^ *vs.* total heart nuclei (values are normalized to C; n = 3 at each time point; *** *p*<0.001 *vs.* C). These three assays show a peak of cardiac cell apoptosis 14–18 h after H/R.
(TIFF)

Figure S5 **Masson Trichrome Staining.** (a–f) Masson trichrome staining in control (C), at 3 h and 30d after H/R. (n = 3) (a–c) calibration bar = 100 µm, (d–f) calibration bar = 50 µm.
(TIF)

Table S1 **Raw data of DNA fragmentation, Caspase-3^+^ and TUNEL^+^ cells experiment.** Table shows DNA fragmentation (a), Caspase-3 (b) and TUNEL^+^ cells (c) raw data. (a) Raw data of DNA fragmentation in control (C) and at different time points (14 h, 18 h and 24 h) after H/R (C, n = 4; 14 h and 18 h, n = 5; 24 h, n = 4 ). (b) Raw data of Caspase-3^+^ cells in control (C) and at different time points (6 h, 14 h, 18 h and 24 h) after H/R (n = 3 at each time point). (c) Raw data of TUNEL^+^ cells in control (C) and at different time points (14 h, 18 h and 24 h) after H/R (n = 3 at each time point).
(DOCX)

Table S2 **DA, SA and FAC values measured by 2D-echocardiography.** Table shows SA, DA and FAC measurements under control conditions (C), 18 h and 30d after H/R. Significant increase in SA and decrease in FAC were observed 18 h after H/R; both SA and FAC exhibited a full recovery by the 30d time point (n = 12 in each group; the same animals were used at each time point).
(DOCX)

Data S1 **Supporting methods.**
(DOCX)

Movie S1 **Representative 2D-echocardiography movie of a control zebrafish heart.**
(WMV)

Movie S2 **Representative 2D-echocardiography movie of a treated zebrafish heart 18** **h after H/R.**
(WMV)

Movie S3 **Representative 2D-echocardiography movie of a treated zebrafish heart 30d after H/R.**
(WMV)
