# Supplementary figures and images for: Hypoxia/Reoxygenation Cardiac Injury and Regeneration in Zebrafish Adult Heart
Source: PLoS One. 2013 Jan 16;8(1):e53748. doi: 10.1371/journal.pone.0053748 (PMC3547061; doi:10.1371/journal.pone.0053748)

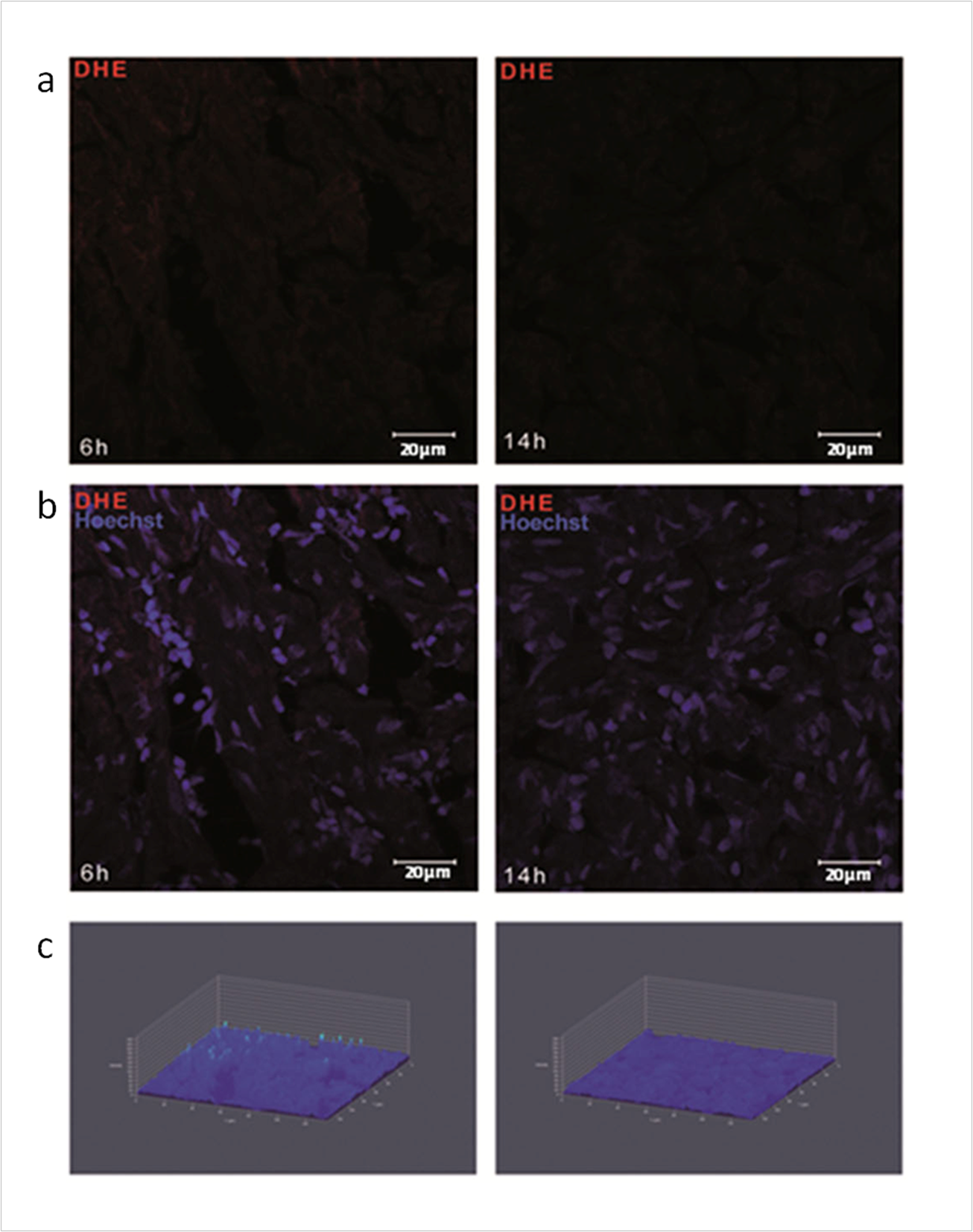

Supplement: Figure S1 — Oxidative stress detection by DHE fluorescence after H/R in vivo. (a) Representative confocal microscopy images of DHE staining at 6 and 14 h after H/R. (b) Merge of DHE and Hoechst nuclear staining. Calibration bar = 20 µm. White arrow-heads indicate DHE+ nuclei. (c) 3D representation of DHE fluorescence intensity distribution in the analyzed area: the z-axis shows the fluorescence intensity in cardiac nuclei, the y-axis and x-axis show the spatial distribution of nuclei on a plane. (TIF) [file pone.0053748.s001.tif]

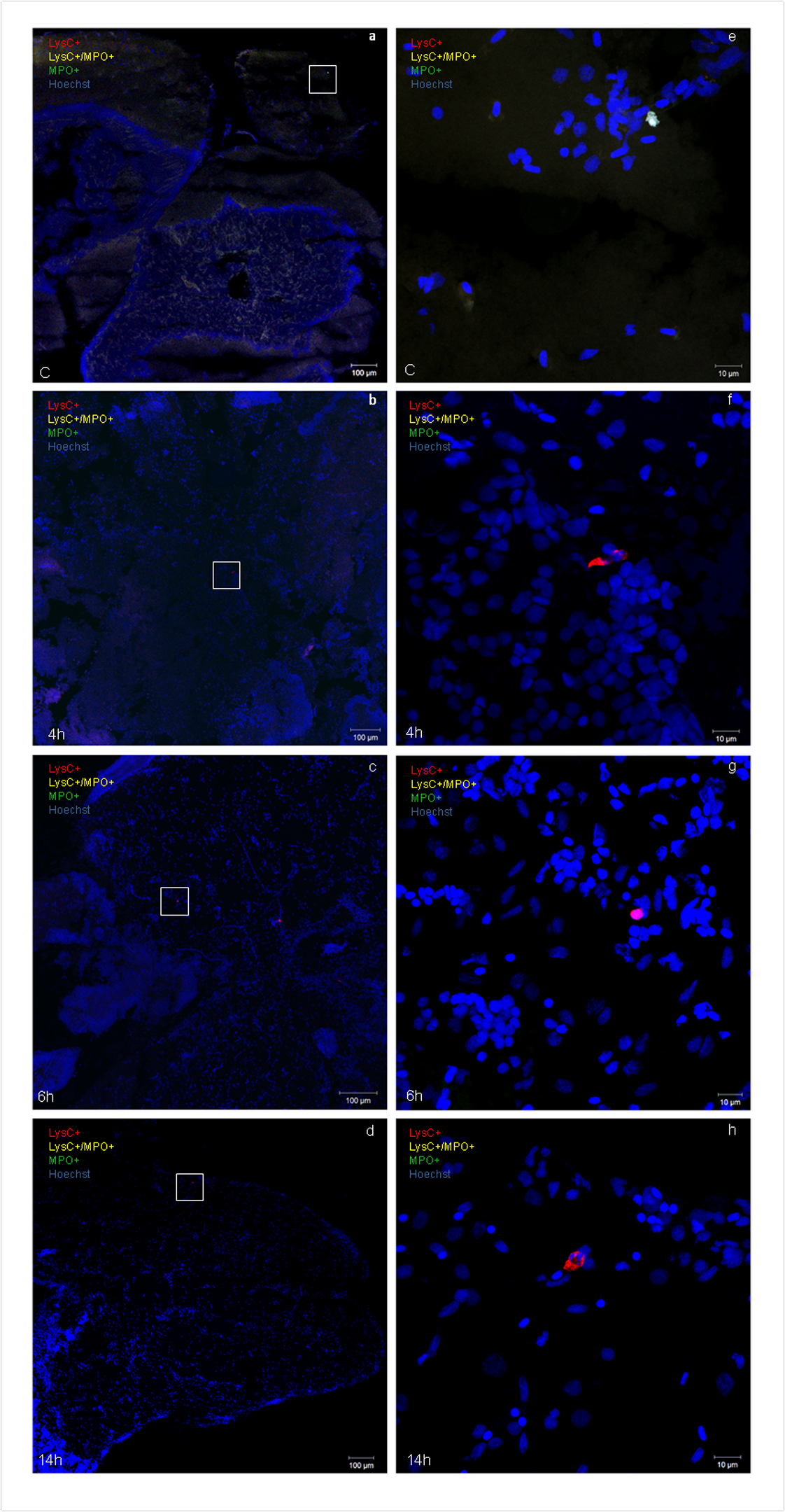

Supplement: Figure S2 — Brain inflammatory response induced by H/R in vivo. (a–h) Representative confocal microscopy images showing neutrophils (yellow or green fluorescence) and macrophages (red fluorescence) infiltration in double transgenic line Tg(MPO:EGFP)×Tg(LysC:DsRed) in control (C) and at different time points (4 h, 6 h, and 14 h) after H/R. Hoechst stains cell nuclei. (n = 3). (a–d) calibration bar = 100 µm, (e–h) calibration bar = 10 µm. (TIF) [file pone.0053748.s002.tif]

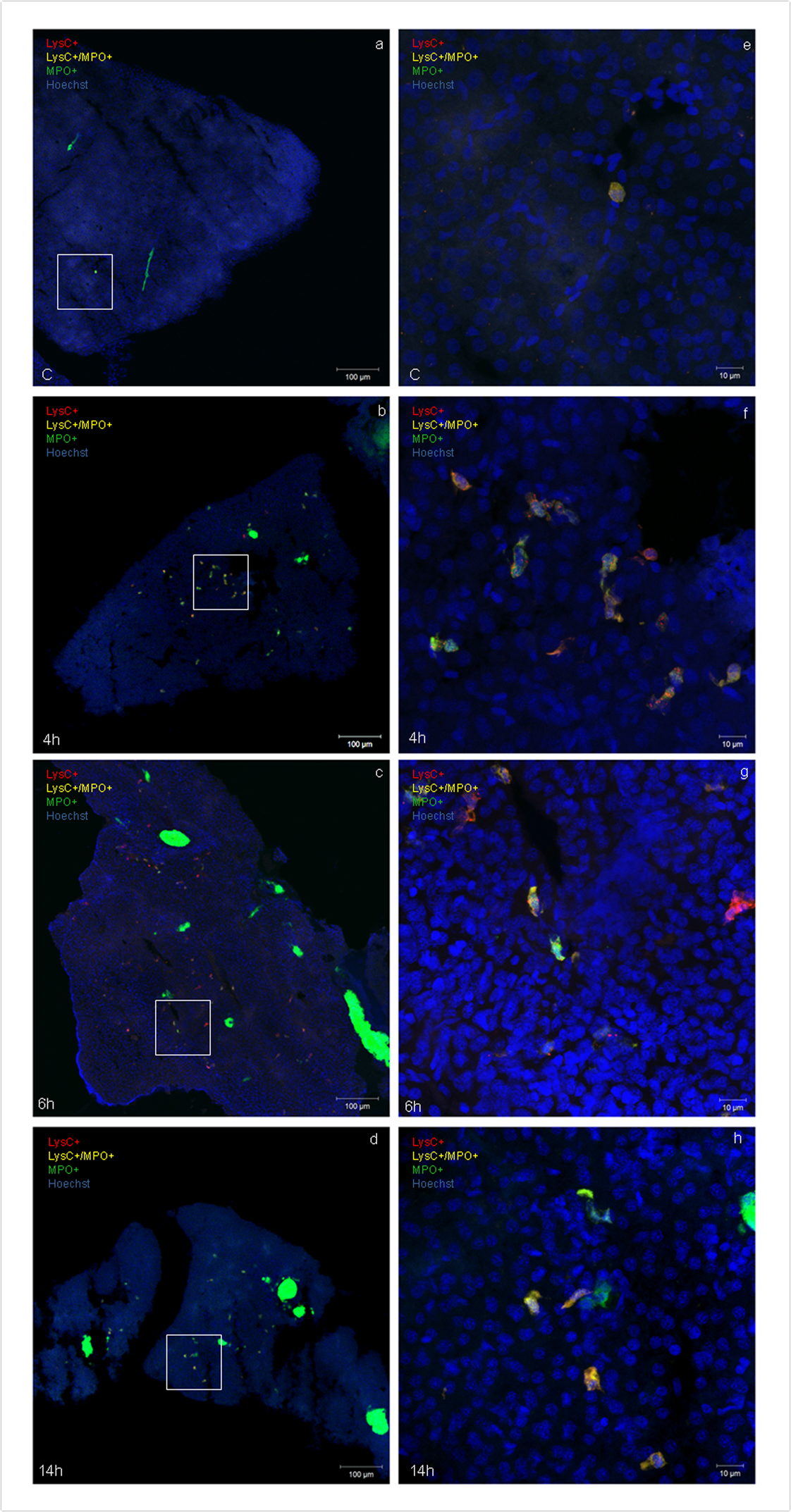

Supplement: Figure S3 — Liver inflammatory response induced by H/R in vivo. (a–h) Representative confocal microscopy images showing neutrophils (yellow or green fluorescence) and macrophages (red fluorescence) infiltration in double transgenic line Tg(MPO:EGFP)×Tg(LysC:DsRed) in control (C) and at different time points (4 h, 6 h, and 14 h) after H/R. Hoechst stains cell nuclei. (n = 3). (a–d) calibration bar = 100 µm, (e–h) calibration bar = 10 µm. (TIF) [file pone.0053748.s003.tif]

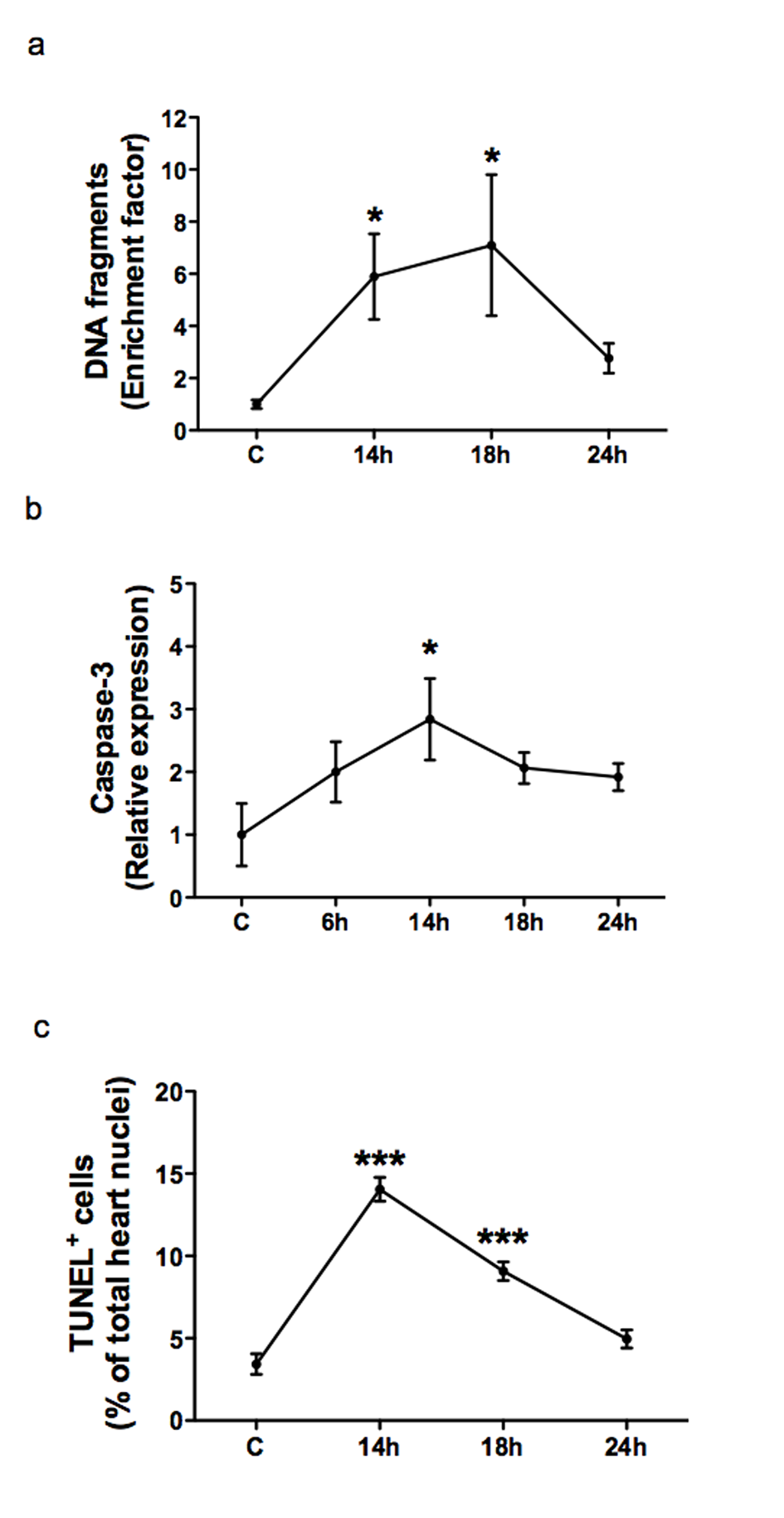

Supplement: Figure S4 — Cardiac cell apoptosis induced by H/R in vivo. (a) ELISA-based quantitation of cytoplasmic oligonucleosome-bound DNA in zebrafish whole heart lysates. The graph shows apoptosis in control (C) and at different time points (14 h, 18 h, and 24 h) after H/R (values are normalized to C; n = 4 at each time point; * p<0.05 vs. C). Enrichment factor is measured as absorbance of treated heart vs. absorbance of control heart. (b) Western blot analysis of caspase-3 activation in whole single zebrafish heart lysates in C and 6 h to 24 h after H/R (values are normalized to C; n = 3 at each time point; * p<0.05 vs. C). Relative expression is referred to densitometric analysis data. (c) DNA fragmentation by TUNEL staining of paraffin embedded heart sections. The graph shows apoptosis in C and 14 h to 24 h after H/R. Data are expressed as percentage of TUNEL+ vs. total heart nuclei (values are normalized to C; n = 3 at each time point; *** p<0.001 vs. C). These three assays show a peak of cardiac cell apoptosis 14–18 h after H/R. (TIFF) [file pone.0053748.s004.tiff]

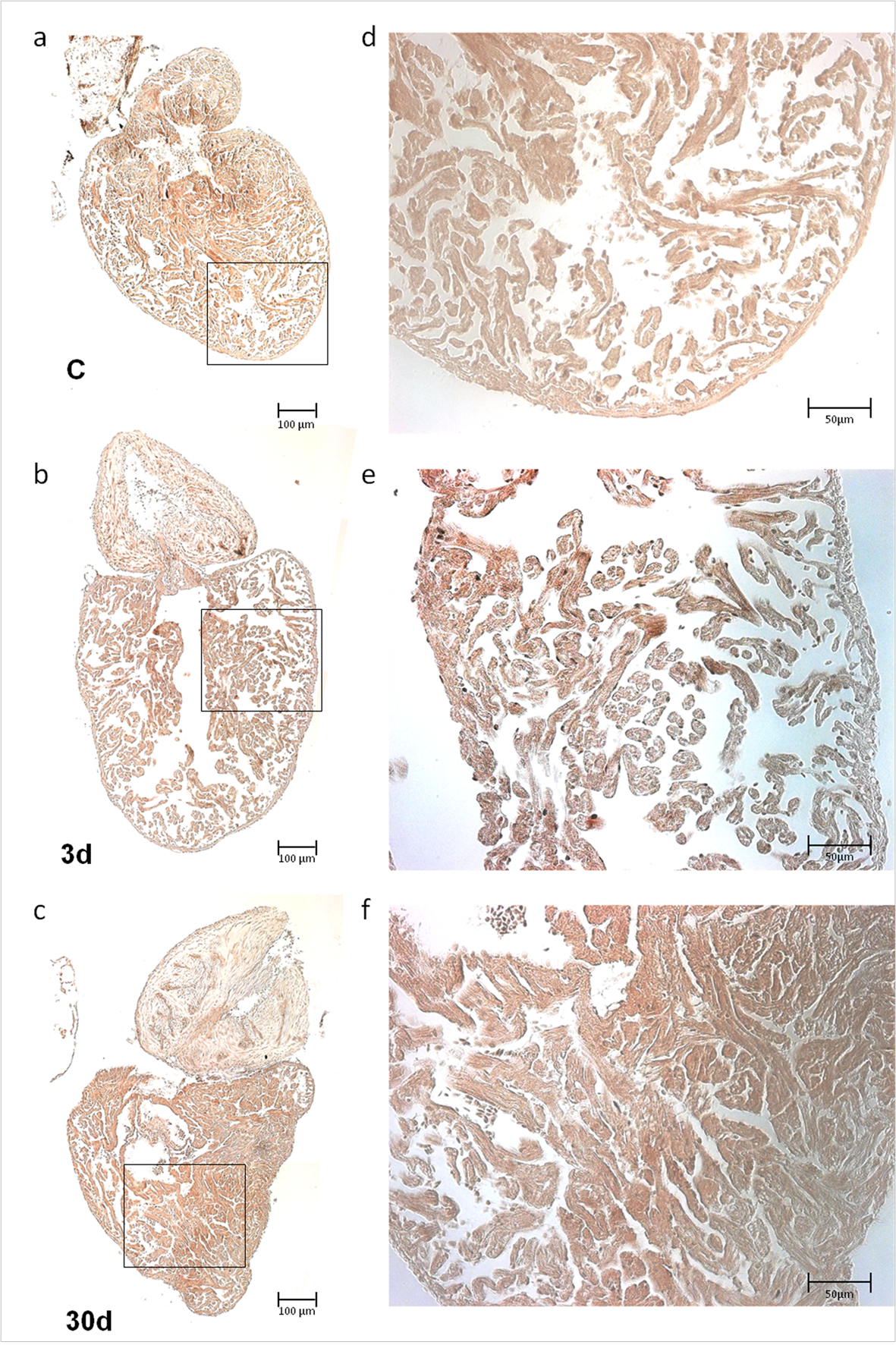

Supplement: Figure S5 — Masson Trichrome Staining. (a–f) Masson trichrome staining in control (C), at 3 h and 30d after H/R. (n = 3) (a–c) calibration bar = 100 µm, (d–f) calibration bar = 50 µm. (TIF) [file pone.0053748.s005.tif]
