# Supplementary material for: Hypoxia/Reoxygenation Cardiac Injury and Regeneration in Zebrafish Adult Heart
Source: PLoS One. 2013 Jan 16;8(1):e53748. doi: 10.1371/journal.pone.0053748 (PMC3547061; doi:10.1371/journal.pone.0053748)
Supplement: Table S1 — Raw data of DNA fragmentation, Caspase-3+ and TUNEL+ cells experiment. Table shows DNA fragmentation (a), Caspase-3 (b) and TUNEL+ cells (c) raw data. (a) Raw data of DNA fragmentation in control (C) and at different time points (14 h, 18 h and 24 h) after H/R (C, n = 4; 14 h and 18 h, n = 5; 24 h, n = 4 ). (b) Raw data of Caspase-3+ cells in control (C) and at different time points (6 h, 14 h, 18 h and 24 h) after H/R (n = 3 at each time point). (c) Raw data of TUNEL+ cells in control (C) and at different time points (14 h, 18 h and 24 h) after H/R (n = 3 at each time point). (DOCX) [file pone.0053748.s006.docx]

**Table S1**

**Raw data of DNA fragmentation, Caspase-3^+^ and TUNEL^+^ cells experiment**

Table shows DNA fragmentation (a), Caspase-3 (b) and TUNEL^+^ cells (c) raw data.

(a) Raw data of DNA fragmentation in control (C) and at different time points (14h, 18h and 24h) after H/R (C, n=4; 14h and 18h, n=5; 24h, n=4 ).

(b) Raw data of Caspase-3^+^ cells in control (C) and at different time points (6h, 14h, 18h and 24h) after H/R (n=3 at each time point).

(c) Raw data of TUNEL^+^ cells in control (C) and at different time points (14h, 18h and 24h) after H/R (n=3 at each time point).

|  | **DNA fragments** | | | |  |
| --- | --- | --- | --- | --- | --- |
| **a** | **C** | **14hr** | **18hr** | **24hr** |  |
|  | 0.77 | 5.13 | 13.65 | 3.14 |  |
|  | 0.79 | 3.65 | 1.3 | 1.18 |  |
|  | 0.95 | 1.91 | 13.37 | 2.88 |  |
|  | 1.49 | 11.38 | 1.9 | 3.87 |  |
|  |  | 7.41 | 5.26 |  |  |
|  |  |  |  |  |  |
|  |  |  |  |  |  |
|  | **Caspase-3** | | | | |
| **b** | **C** | **6hr** | **14hr** | **18hr** | **24hr** |
|  | 0.17 | 1.15 | 2.25 | 2 | 1.84 |
|  | 0.94 | 2.03 | 2.13 | 1.67 | 1.59 |
|  | 1.89 | 2.82 | 4.14 | 2.52 | 2.33 |
|  |  |  |  |  |  |
|  |  |  |  |  |  |
|  | **TUNEL^+^ cells** | | | |  |
| **c** | **C** | **14hr** | **18hr** | **24hr** |  |
|  | 2.42 | 13.57 | 10.09 | 4.53 |  |
|  | 4.56 | 13.1 | 8.12 | 6.05 |  |
|  | 3.31 | 15.47 | 9.02 | 4.3 |  |
