# Supplementary material for: Hypoxia/Reoxygenation Cardiac Injury and Regeneration in Zebrafish Adult Heart
Source: PLoS One. 2013 Jan 16;8(1):e53748. doi: 10.1371/journal.pone.0053748 (PMC3547061; doi:10.1371/journal.pone.0053748)
Supplement: Table S2 — DA, SA and FAC values measured by 2D-echocardiography. Table shows SA, DA and FAC measurements under control conditions (C), 18 h and 30d after H/R. Significant increase in SA and decrease in FAC were observed 18 h after H/R; both SA and FAC exhibited a full recovery by the 30d time point (n = 12 in each group; the same animals were used at each time point). (DOCX) [file pone.0053748.s007.docx]

**Table S2**

**DA, SA and FAC values measured by 2D-echocardiography**

Table shows SA, DA and FAC measurements under control conditions (C), 18h and 30d after H/R. Significant increase in SA and decrease in FAC were observed 18h after H/R; both SA and FAC exhibited a full recovery by the 30d time point (n=12 in each group; the same animals were used at each time point).

|  | **C** | **18h** | **30d** |
| --- | --- | --- | --- |
| **SA** | 0.739±0.037 | 0.956±0.067** | 0.698±0.039 |
| **DA** | 1.048±0.050 | 1.140±0.068 | 0.974±0.042 |
| **FAC** | 29.3±2.0% | 16.4±1.8%*** | 28.6±1.6% |

***p* < 0.01 and *** *p* < 0.001 *vs.* C
