# Supplementary material for: Hypoxia/Reoxygenation Cardiac Injury and Regeneration in Zebrafish Adult Heart
Source: PLoS One. 2013 Jan 16;8(1):e53748. doi: 10.1371/journal.pone.0053748 (PMC3547061; doi:10.1371/journal.pone.0053748)
Supplement: Data S1 — Supporting methods. (DOCX) [file pone.0053748.s008.docx]

**SUPPLEMENTARY DATA**

**SUPPLEMENTARY METHODS**

**Measurement of intracellular reactive oxygen species after H/R**

To perform DHE staining, cryosections were incubated with DHE (5 mM in Ringer’s Solution, Ca^2+^ free) for 60 min at 37°C in a light-protected humidity chamber, then fixed with 4% PBS buffered-paraformaldehyde (PFA; Sigma-Aldrich, St Louis, MO) and counterstained with Hoechst 33258 (Sigma-Aldrich). Slides were then mounted and imaged by confocal microscopy (LSM 710; Carl Zeiss, Jena, Germany). The analysis was performed with Zen Software 2008 (Carl Zeiss); fluorescence was quantified by counting the pixels in correspondent image fields in each group, pixels intensity was then quantified and finally expressed as mean fluorescence intensity (MFI).

To assay proteins nitrosylation, cryosections were 4% PFA-fixed, permeabilized with Cytonin-IHC™ (Trevigen, Gaithersburg, MD) and incubated overnight with the primary antibody. We used the goat anti-rabbit Alexa Fluor 488 (Invitrogen) as secondary antibody, and sections were finally Hoechst 33258 counterstained. Imaging and fluorescence quantification was performed as described above.

**HIF-1α – dependent genes expression and determination of cardiac cell apoptosis**

Total RNA was isolated from whole adult heart using the RNAqueous–Micro kit (Ambion, Austin, TX) and the TissueLyser homogenizer (Qiagen, Hilden, Germany). Genomic DNA was removed by Turbo DNA-free DNase (Ambion) digestion. One µg of total RNA was reverse-transcribed using the High Capacity cDNA Reverse Transcription Kit (Applied Biosystems, Forster City, CA). A custom TaqMan Array was designed to assess the expression of Heme-oxygenase (decycling) 1 (*hmox1*), vascular endothelial growth factor Aa (*vegfaa*), and erythropoietin (*epo*), using a 7900HT Fast Real-Time PCR System (Applied Biosystems). A set of reference genes, i.e. eukaryotic 18S rRNA (*18S*), eukaryotic translation elongation factor 1 gamma (*eef1g*) and glyceraldehyde-3-phosphate dehydrogenase (*gapdh*), was included for normalization of the raw expression intensities of target mRNAs. Data were analyzed with the software ABI RQ Manager 1.2.1, setting the threshold for cycle threshold (Ct) manually at the same value, to allow comparison of multiple arrays. All Ct values reported as greater than 40 or as not detected were changed to 40 and considered negative calls. Analysis of gene expression stability and selection of the best reference gene (*eef1g*) were performed with the NormFinder 0.953 Excel Add-In.[35] The relative expression of any target gene, normalized to the endogenous reference and relative to the calibrator (mean value of the control untreated samples), was calculated using the comparative Ct method.[36]

Three independent methods were used to evaluate cardiac cell apoptosis:

-detection of cytosolic oligonucleosome-bound DNA; DNA fragmentation was measured by quantitation of cytosolic oligonucleosome-bound DNA using the Cell Death Detection ELISA kit (Roche, Basel, Switzerland), according to the manufacturer’s instructions. Briefly, the cytosolic fraction (isolated at 13.000 × *g*) of whole heart homogenates was used as antigen source in a sandwich ELISA with a primary anti-histone antibody coated to the microtiter plate, and an anti-DNA antibody coupled to peroxidase. Absorbance was measured at 405 nm. Enrichment of mono- and oligo-nucleosomes released into the cytoplasm was calculated using the following formula: Enrichment factor = absorbance of the sample (treated heart) / absorbance of the control (untreated heart).

-quantitation of caspase-3 activity; whole hearts from untreated and H/R adult zebrafish were homogenized (TissueLyser, Qiagen) in Laemmli buffer (100 mM Tris, pH 6.8, 20% glycerol, 4% SDS, with protease inhibitors cocktail). Immunoblottings were performed according to standard procedures. Cardiac lysates were precipitated at 13.000 × *g* and protein concentrations were determined. Protein extracts (50 μg per lane) were heat-denatured at 95°C and loaded on a 12% SDS-polyacrylamide gel. An anti-active caspase-3 polyclonal antibody (Chemicon) or anti-acetylated tubulin monoclonal antibody (Sigma-Aldrich) were used as primary antibodies: detection was carried out on protein samples transferred to nitrocellulose membrane. Membranes were blocked (4% BSA/TBS) and then incubated overnight at 4°C with the primary antibody 1:100 in 4% BSA/TBS. Goat anti-rabbit or anti-mouse peroxidase-coupled antibodies were used as secondary antibodies. Detection was carried out with SuperSignal West Dura Extended Duration Chemiluminescent Substrate (Thermo Scientific) and the proteins were visualized on autoradiographic films (Kodak BioMax MR Film; Sigma-Aldrich). Densitometric analysis was performed by Quantity One 1-D Analysis Software (GelDoc 2000; Bio-Rad Laboratories, Hercules, CA), in order to perform protein quantitation. Acetylated tubulin was used as protein loading control.

-*in situ* detection of DNA fragmentation; normoxic and hypoxic adult zebrafish hearts were fixed in 4% PFA immediately after explantation and processed for paraffin embedding. Sections (5µm) were cut and CardioTACS^TM^ In Situ Apoptosis Detection Kit (Trevigen) was used to assess nuclear DNA fragmentation *in situ* by terminal deoxy-nucleotidyl transferase-mediated dUTP nick end-labelling (TUNEL) according to manufacturer’s protocol. For each section, the number of TUNEL^+^ nuclei was scored in ten different microscope fields (1200-2000 nuclei per heart). Sections were imaged by light microscopy (AxioSkop 1-Plus; Carl Zeiss) and digital pictures were analyzed with the AxioVision image analysis software (Carl Zeiss). Apoptotic index was expressed as the percentage of TUNEL^+^ nuclei *vs* the total number of nuclei previously counterstained with Nuclear Fast Red (Trevigen).

**Determination of myocardial cell death and proliferation**

In order to analyze cardiomyocyte apoptosis, 8µm cryosections from transgenic Tg(cmlc2:nucDsRed) untreated or H/R hearts were stained with the In Situ Cell Death Detection Kit, Fluorescein (Roche Applied Science), following manufacturer's protocol. Fluorescein signal was amplified with anti Fluorescein/Oregon green Alexa Fluor 488 conjugated antibody (Invitrogen), and total nuclei were counterstained with DAPI (Sigma-Aldrich). Slides were then mounted and imaged by confocal microscopy (LSM 710; Carl Zeiss). Cardiomyocytes nuclei were detected as DsRed^+^ cells, and the apoptotic index was expressed as the percentage of TUNEL^+^ cardiomyocytes nuclei *vs.* total DsRed^+^ cardiomyocytes nuclei (using the Zen image analysis software, Carl Zeiss, and the Image J software, NIH).

Myocardial cell necrosis was evaluated by Acridine Orange (AO) staining[5, 10, 11]. Zebrafish hearts were incubated in AO/PBS (2 µg/mL; Sigma-Aldrich) for 1h, washed in PBS for 10 min, OCT-embedded and cryosectioned. The resulting 8µm cryosections were 4% PFA-fixed and total nuclei were counterstained with DAPI (Sigma) before mounting. Slides were then mounted and imaged by confocal microscopy (LSM 710; Carl Zeiss). Cardiomyocytes nuclei were detected as DsRed^+^ cells, and the necrotic index was expressed as the percentage of AO^+^ cardiomyocytes nuclei *vs.* total DsRed^+^ cardiomyocytes nuclei (using the Zen and the Image J software).

Cardiomyocytes proliferation was assayed in untreated and H/R hearts from Tg(cmlc2:nucDsRed) zebrafish line by immunofluorescence staining with Phospho Histone H3 (pHH3) and Proliferating Cell Nuclear Antigen (PCNA) markers. For pHH3 immunostaining, 8µm cryosections were 4% PFA-fixed, permeabilized with 0.2% PBTr, non-specific binding sites were saturated for 1h in blocking solution (10% goat serum) and sections were incubated overnight with the primary antibody 1:200 (rabbit polyclonal antibody, Bethyl, Montgomery TX); AlexaFluor 488 goat anti-rabbit (Invitrogen) was used as secondary antibody 1:200, sections were DAPI counterstained before mounting. For PCNA immunostaining, 5µm paraffin embedded sections were deparaffinized, rehydrated and washed in distilled water; antigen retrieval was performed in citrate buffer (pH 6.0) for 45 min in water bath at 95°C. Non-specific binding sites were saturated for 1h in blocking solution (10% goat serum), sections were incubated overnight with anti-PCNA 1:100 (rabbit polyclonal antibody, Santa Cruz Biotechnology, Santa Cruz, CA); FITC goat anti-rabbit 1:50 (Invitrogen) was used as secondary antibody, and the signal was amplified by rabbit anti-FITC 1:500 (Invitrogen) and AlexaFluor 488 goat anti-rabbit (Invitrogen). DsRed in cmlc2 nuclei was recovered by rabbit anti-DsRed 1:100 (Invitrogen) and AlexaFluor 594 goat anti-rabbit (Invitrogen) as secondary antibody 1:200. Sections were DAPI counterstained before mounting. Slides were imaged by confocal microscopy (LSM 710; Carl Zeiss). Cardiomyocytes nuclei were detected as DsRed^+^ cells, and the proliferation index was expressed as the percentage of pHH3^+^ or PCNA^+^ cardiomyocytes nuclei *vs* total DsRed^+^ cardiomyocytes nuclei (using the Zen and the Image J software).

**Quantification of cardiomyocyte death and proliferation**

For cell death and proliferation, the medial longitudinal section of each ventricle was selected for quantification, and high-magnification images were taken of different ventricular regions and merged using Adobe Photoshop software to represent the whole ventricular section. DsRed^+^ nuclei (500-1200 per animal) were counted manually using Image J software (NIH), to identify cardiomyocytes positive for AO, TUNEL, pHH3 or PCNA.

The presence of connective tissue was evaluated by Masson trichrome staining (Bio-Optica, Italy).

**SUPPLEMENTARY FIGURES**

**Figure S1. Oxidative stress detection by DHE fluorescence after H/R *in vivo***

(a) Representative confocal microscopy images of DHE staining at 6 and 14h after H/R. (b) Merge of DHE and Hoechst nuclear staining. Calibration bar = 20 µm. White arrow-heads indicate DHE^+^ nuclei. (c) 3D representation of DHE fluorescence intensity distribution in the analyzed area: the *z-axis* shows the fluorescence intensity in cardiac nuclei, the *y-axis* and *x-axis* show the spatial distribution of nuclei on a plane.

**Figure S2. Brain inflammatory response induced by H/R *in vivo***

(a-h) Representative confocal microscopy images showing neutrophils (yellow or green fluorescence) and macrophages (red fluorescence) infiltration in double transgenic line Tg(MPO:EGFP) x Tg(LysC:DsRed) in control (C) and at different time points (4h, 6h, and 14h) after H/R. Hoechst stains cell nuclei. (n=3). (a-d) calibration bar = 100 µm, (e-h) calibration bar = 10 µm.

**Figure S3. Liver inflammatory response induced by H/R *in vivo***

(a-h) Representative confocal microscopy images showing neutrophils (yellow or green fluorescence) and macrophages (red fluorescence) infiltration in double transgenic line Tg(MPO:EGFP) x Tg(LysC:DsRed) in control (C) and at different time points (4h, 6h, and 14h) after H/R. Hoechst stains cell nuclei. (n=3). (a-d) calibration bar = 100 µm, (e-h) calibration bar = 10 µm.

**Figure S4. Cardiac cell apoptosis induced by H/R *in vivo***

(a) ELISA-based quantitation of cytoplasmic oligonucleosome-bound DNA in zebrafish whole heart lysates. The graph shows apoptosis in control (C) and at different time points (14h, 18h, and 24h) after H/R (values are normalized to C; n=4 at each time point; * *p* < 0.05 *vs.* C). Enrichment factor is measured as absorbance of treated heart *vs.* absorbance of control heart.

(b) Western blot analysis of caspase-3 activation in whole single zebrafish heart lysates in C and 6h to 24h after H/R (values are normalized to C; n=3 at each time point; * *p* < 0.05 *vs.* C). Relative expression is referred to densitometric analysis data.

(c) DNA fragmentation by TUNEL staining of paraffin embedded heart sections. The graph shows apoptosis in C and 14h to 24h after H/R. Data are expressed as percentage of TUNEL^+^ *vs.* total heart nuclei (values are normalized to C; n=3 at each time point; *** *p* < 0.001 *vs.* C).

These three assays show a peak of cardiac cell apoptosis 14-18h after H/R.

**Figure S5. Masson Trichrome Staining**

(a-f) Masson trichrome staining in control (C), at 3h and 30d after H/R. (n=3) (a-c) calibration bar = 100 µm, (d-f) calibration bar = 50 µm.

**Table S1**

**Raw data of DNA fragmentation, Caspase-3^+^ and TUNEL^+^ cells experiment**

Table shows DNA fragmentation (a), Caspase-3 (b) and TUNEL^+^ cells (c) raw data.

(a) Raw data of DNA fragmentation in control (C) and at different time points (14h, 18h and 24h) after H/R (C, n=4; 14h and 18h, n=5; 24h, n=4 ).

(b) Raw data of Caspase-3^+^ cells in control (C) and at different time points (6h, 14h, 18h and 24h) after H/R (n=3 at each time point).

(c) Raw data of TUNEL^+^ cells in control (C) and at different time points (14h, 18h and 24h) after H/R (n=3 at each time point).

|  | **DNA fragments** | | | |  |
| --- | --- | --- | --- | --- | --- |
| **a** | **C** | **14hr** | **18hr** | **24hr** |  |
|  | 0.77 | 5.13 | 13.65 | 3.14 |  |
|  | 0.79 | 3.65 | 1.3 | 1.18 |  |
|  | 0.95 | 1.91 | 13.37 | 2.88 |  |
|  | 1.49 | 11.38 | 1.9 | 3.87 |  |
|  |  | 7.41 | 5.26 |  |  |
|  |  |  |  |  |  |
|  |  |  |  |  |  |
|  | **Caspase-3** | | | | |
| **b** | **C** | **6hr** | **14hr** | **18hr** | **24hr** |
|  | 0.17 | 1.15 | 2.25 | 2 | 1.84 |
|  | 0.94 | 2.03 | 2.13 | 1.67 | 1.59 |
|  | 1.89 | 2.82 | 4.14 | 2.52 | 2.33 |
|  |  |  |  |  |  |
|  |  |  |  |  |  |
|  | **TUNEL^+^ cells** | | | |  |
| **c** | **C** | **14hr** | **18hr** | **24hr** |  |
|  | 2.42 | 13.57 | 10.09 | 4.53 |  |
|  | 4.56 | 13.1 | 8.12 | 6.05 |  |
|  | 3.31 | 15.47 | 9.02 | 4.3 |  |

**Table S2**

**DA, SA and FAC values measured by 2D-echocardiography**

Table shows SA, DA and FAC measurements under control conditions (C), 18h and 30d after H/R. Significant increase in SA and decrease in FAC were observed 18h after H/R; both SA and FAC exhibited a full recovery by the 30d time point (n=12 in each group; the same animals were used at each time point).

|  | **C** | **18h** | **30d** |
| --- | --- | --- | --- |
| **SA** | 0.739±0.037 | 0.956±0.067** | 0.698±0.039 |
| **DA** | 1.048±0.050 | 1.140±0.068 | 0.974±0.042 |
| **FAC** | 29.3±2.0% | 16.4±1.8%*** | 28.6±1.6% |

***p* < 0.01 and *** *p* < 0.001 *vs.* C

**Movie S1. Representative 2D-echocardiography movie of a control zebrafish heart**

**Movie S2. Representative 2D-echocardiography movie of a treated zebrafish heart 18h after H/R**

**Movie S3. Representative 2D-echocardiography movie of a treated zebrafish heart 30d after H/R**
